# A Nomogram for Predicting Recurrence in Stage I Non‐Small Cell Lung Cancer

**DOI:** 10.1111/crj.70022

**Published:** 2024-11-24

**Authors:** Rongrong Bian, Feng Zhao, Bo Peng, Jin Zhang, Qixing Mao, Lin Wang, Qiang Chen

**Affiliations:** ^1^ Department of Oncology Nanjing Liuhe District People's Hospital Nanjing China; ^2^ Department of Thoracic Surgery Taixing People's Hospital Taixing China; ^3^ Department of Thoracic Surgery, Xuzhou Central Hospital XuZhou Clinical School of Xuzhou Medical University Xuzhou Jiangsu China; ^4^ Department of Oncology, Department of Geriatric Lung Cancer Laboratory The Affiliated Geriatric Hospital of Nanjing Medical University, Jiangsu Province Geriatric Hospital Nanjing China; ^5^ Department of Thoracic Surgery, Jiangsu Cancer Hospital, Jiangsu Institute of Cancer Research Nanjing Medical University Affiliated Cancer Hospital Nanjing China

**Keywords:** nomogram, recurrence, stage I non–small cell lung cancer, survival

## Abstract

**Background:**

Early‐stage non–small cell lung cancer (NSCLC) is being diagnosed increasingly, and in 30% of diagnosed patients, recurrence will develop within 5 years. Thus, it is urgent to identify recurrence‐related markers to optimize the management of patient‐tailored therapeutics.

**Methods:**

The eligible datasets were downloaded from TCGA and GEO. In the discovery phase, two algorithms, least absolute shrinkage and selector operation and support vector machine‐recursive feature elimination, were used to identify candidate genes. The recurrence‐associated signature was developed by penalized Cox regression. The nomogram was constructed and further tested via other independent cohorts.

**Results:**

In this retrospective study, 14 eligible datasets and 7 published signatures were included. A 13‐gene based signature was generated by penalized Cox regression categorized training cohort into high‐risk and low‐risk subgroups (HR = 8.873, 95% CI: 4.228–18.480 *p* < 0.001). Furthermore, a nomogram integrating the recurrence‐related signature, age, and histology was developed to predict the recurrence‐free survival in the training cohort, which performed well in the two external validation cohorts (concordance index: 0.737, 95% CI: 0.732–0.742, *p* < 0.001; 0.666, 95% CI: 0.650–0.682, *p* < 0.001; 0.651, 95% CI: 0.637–0.665, *p* < 0.001, respectively). The nomogram was further performed well in the Jiangsu cohort enrolled 163 patients (HR = 2.723, 95% CI: 1.526–4.859, *p* = 0.001). Post‐operative adjuvant therapy achieved evaluated disease‐free survival in high and intermediate risk groups (HR = 4.791, 95% CI: 1.081–21.231, *p* = 0.039).

**Conclusions:**

The proposed nomogram is a promising tool for estimating recurrence‐free survival in stage I NSCLC, which might have tremendous value in management of early stage NSCLC and guiding adjuvant therapy strategies.

## Graphical abstract

The aim of the study was to develop a feasible tool to optimize the recurrence prediction of stage I NSCLC. The eligible datasets were downloaded from TCGA and GEO. A 13‐gene based signature was generated by two algorithms. Then, a nomogram integrating the signature, age, and histology was developed and validated to predict the recurrence‐free survival. The nomogram is a promising tool for estimating recurrence‐free survival of stage I NSCLC, which may have potential value in guiding adjuvant therapy.

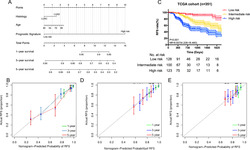

**Abbreviations**
LASSO: least absolute shrinkage and selector operation
NSCLC: non–small cell lung cancer
SVM‐RFE: support vector machine‐recursive feature elimination

## 1 Introduction

With the adoption of low‐dose spiral computed tomography screening for the high‐risk individuals, the proportion of stage I non‐small cell lung cancer (NSCLC) rise sharply. Surgical resection is the curative treatment for stage I NSCLC. However, local relapse and metastasis impede the therapeutic effect, and approximately 30% patients will suffer recurrence within 5 years of diagnosis [1, 2]. Postoperative adjuvant therapy is a valid approach to prevent the relapse and metastasis, but the effect is still unclear in stage I NSCLC [3, 4]. Several large randomized studies failed to show a survival benefit from stage I NSCLC patients who received adjuvant systemic treatment after surgery due to side effects of therapy [5]. Thus, in view of the high rate of relapse and the lack of useful biomarkers, a feasible prediction model is needed to predict the relapse‐free survival of stage I NSCLC patients and identify the high risk patients who may benefit from postoperative adjuvant therapy.

Plenty of studies have been published to identify clinical risk factors for survival or relapse [6]. However, it is not enough to predict the relapse by only considering clinical risk factors, because stage I NSCLC is a heterogeneous disease, which comprising different subgroups with distinct molecular alterations [7]. Expression profiling has showed great promise in getting insight of molecular mechanisms and biomarkers through analysis of thousands of genes [8, 9]. And increasing molecular markers have been incorporated into clinical decision‐making [10]. Previous studies highlighted several relapse‐related signatures for stage I NSCLC, which showed molecular information and provided a more robust biomarker of relapse [11, 12, 13]. Unfortunately, overfitting on small discovery data sets and lack of sufficient external validation impeded them to be widespread adopted into clinical practice. Therefore, developing and testing a relapse‐related signature in a large‐scale study is needed, and available public gene expression data sets with relapse status brings the opportunity to identify more reliable signature for relapse. In addition, there is an increasing trend that combining molecular features and clinicopathologic features to predict the disease status or prognosis by recent investigations [14].

In this study, we aimed to develop and validate a predicting signature related to the relapse by multiple gene expression datasets and leverage the clinical features to build a nomogram predicting the relapse of stage I NSCLC, which would improve capacity of relapse prediction and might have tremendous value in guiding the management of stage I NSCLC patients.

## 2 Method

### 2.1 Study Population and Study Design

Comprehensive search for eligible expression profile datasets related to recurrence of NSCLC was performed in Gene Expression Omnibus (GEO) and The Cancer Genome Atlas (TCGA). Datasets were filtered by the criteria such as containing stage I NSCLC patients, recurrence status, and relapse‐free survival time. After primary filtration, we excluded those patients who had received neoadjuvant therapy, adjuvant chemotherapy, or other pharmaceutical therapy in each dataset. Tumors were pathologically classified as stage I according to the eighth edition of the TNM classification. After removing patients with no clinical or subtype information, demographic and clinical characteristics of the patients were recorded. Simultaneously, searching for published prognostic signatures related to recurrence were also conducted in Pubmed, Embase, and MEDLINE with the key words including “relapse NSCLC,” “recurrence NSCLC,” “relapse stage I NSCLC,” “recurrence stage I NSCLC,” “relapse lung cancer,” “recurrence lung cancer,” “relapse stage I lung cancer,” and “recurrence stage I lung cancer.” Additionally, stage IA NSCLC who underwent radical resection (i.e., lobectomy/sub‐lobectomy and lymph node dissection) were enrolled into Jiangsu (JS) cohort over the years 2014–2016 at the Jiangsu Geriatric Hospital and Xuzhou Central Hospital. Inclusive criteria included (1) all resected cancer tissues were confirmed by pathology, (2) well‐preserved fresh tissues were available, and (3) detailed clinical information and regular follow‐up were included. Progression‐free survival was identified from the radical resection to local recurrence or metastasis detected by radiological examination. Regular follow‐up included chest CT scan routinely every 3 months and contrast‐enhanced magnetic resonance imaging of head and radionuclide bone scanning annually after surgery. Ethics committee of the Jiangsu Geriatric Hospital and Xuzhou Central Hospital (No. 2022009) and informed consent were obtained from all patients.

**FIGURE 1:**
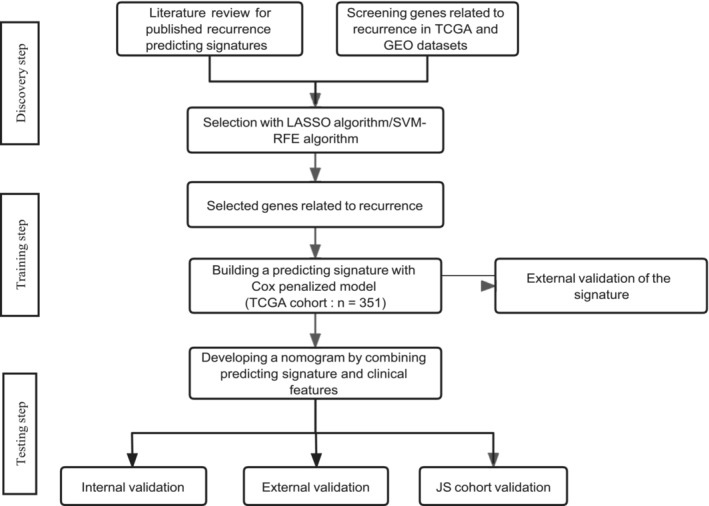
Study flowchart. LASSO, least absolute shrinkage and selector operation; SVM‐RFE, support vector machine‐recursive feature elimination; TCGA, The Cancer Genome Atlas.

Three steps were designed to develop and test the recurrence‐related nomogram for NSCLC patients with stage I. In the discovery phase, eligible datasets and published prognostic signatures were used to screen significant genes related to recurrence by two algorithms (the least absolute shrinkage and selector operation [LASSO] and support vector machine‐recursive feature elimination [SVM‐RFE]), which obtained the candidate features for the further selection. In the training phase, patients from TCGA were included in training cohort due to the completed clinical records and respectively large sample size. Penalized Cox regression was performed to develop a recurrence signature in training cohort, which was subsequently tested in two independent cohorts. Then, a nomogram was constructed by combining recurrence signature and significant clinical features. In the validation phase, two independent GEO datasets and one local cohort were selected to test the performance of the nomogram. The study design was displayed by a flow chat in Figure 1. Recurrence‐free survival was defined as the time from the date of diagnosis to the date of recurrence or last follow‐up. The schedule of follow‐up and examination was described by the corresponding studies. Forest plot analyses were performed using R package, “meta” (https://github.com/guido‐s/meta http://meta‐analysis‐with‐r.org). A heterogeneity test for the combined HR was carried out using the *I* ^2^ statistic.

### 2.2 Statistical Analysis

The expression profile datasets were normalized and qualified using the R package (“affy”). Gene IDs were mapped to genes using the corresponding annotation files. Multiple probes corresponding to the same gene ID were averaged to one measurement for each sample. Differential expression genes between recurrence samples and non‐recurrence samples were generated by “limma” package of R software with adjusted false discovery rate less than 0.05 and fold‐change greater than 2. Recurrence‐related genes were identified by Cox univariable analysis with significant *p* values. Significant genes from each dataset and genes from these prognostic signatures were submitted to the LASSO algorithm in order to select candidate features with optimal lambda value, which was defined by 10‐fold cross‐validation (R package, glmnet). Meanwhile, another method called support vector machine‐recursive feature elimination (SVM‐RFE) was used for selection (R package, e1071). Then, a recurrence‐related signature was developed from all candidate features selected by two algorithms using penalized Cox analysis. The nomogram was developed by “rms” package of R software. The variable included into nomogram were identified by backward wise step method and the clinical significance. The concordance index (C‐index) were used to evaluate the discrimination of the nomogram, and calibration plots revealed the accuracy of the nomogram. “SurvivalROC” package of R software was used to draw the receiver operating characteristic (ROC) curves. All steps were conducted in R version 3.3.3 software. The cut‐off values were defined by X‐tile software with the highest *χ* ^2^ value in each set [15].

## 3 Results

### 3.1 Characteristics of Patients

Retrospectively analysis of the gene expression profiles related to recurrence identified 14 eligible cohorts, including 13 microarray datasets from GEO (https://www.ncbi.nlm.nih.gov/geo/) and 1 RNA‐Seq dataset from The Cancer Genome Atlas (TCGA) (https://cancergenome.nih.gov/). A total of 1414 stage I NSCLC patients were included from 14 published cohorts retrieved by the systematic search. The characteristics of these datasets were listed in Table 1. We did not integrate these datasets together due to different platforms and different racial groups and segregated them into three phrases. TCGA cohort with the largest sample size and complete clinical records were assigned into the training phase, and we selected another two individual datasets with moderate sample size and matching clinical records for independent validation, namely, GEO50081 and GEO30219. In addition, seven published prognostic signatures related to recurrence were identified, which were also incorporated into the discovery phase (Table S1).

**TABLE 1:** Baseline information of TCGA and GEO datasets.

Datasets	Sample size	Platform	No. of stage I	Year	Country
Discovery step					
GSE41271	275	Illumina HumanWG‐6 v3.0 Expression Beadchip	132	2013	USA
GSE7339	100	Hitachisoft AceGene Human Oligo Chip 30K Subset A	—	2007	Japan
GSE11969	163	Agilent *Homo sapiens* 21.6K Custom Array	78	2009	Japan
GSE13213	117	Agilent‐014850 Whole Human Genome Microarray	61	2009	Japan
GSE5843	48	PRHU05‐S1‐0006 (PC Human Operon v2 21k)	48	2007	Australia
GSE8894	138	Affymetrix Human Genome U133 Plus 2.0 Array	88	2007	South Korea
GSE5123	51	GPL3877 PRHU05‐S1‐0006 (PC Human Operon v2 21k)	30	2006	Australia
GSE32863	116	Illumina HumanWG‐6 v3.0 Expression Beadchip	34	2012	USA
GSE68465	462	GPL96 Affymetrix Human Genome U133 Plus 2.0 Array	114	2015	USA
GSE31210	246	GPL570 Affymetrix Human Genome U133 Plus 2.0 Array	162	2011	Japan
GSE37745	196	GPL570 Affymetrix Human Genome U133 Plus 2.0 Array	50	2012	Sweden
Training step					
TCGA	1124	Illumina Hiseq	351	2015	TCGA project
Testing step					
GSE50081	181	GPL570 Affymetrix Human Genome U133 Plus 2.0 Array	124	2013	Canada
GSE30219	307	GPL570 Affymetrix Human Genome U133 Plus 2.0 Array	142	2013	France

The median recurrence‐free survival time of the patients in TCGA was 508 days (range, 2 to 6812 days). During follow‐up, 19.9% of the patients (70 of 351) developed recurrence. The median follow‐up time of the patients in the testing cohort were 1729 days and 1935 days (range, 44 and 4393 days and 30 days and 7680 days, respectively). 25% and 24.6% of the patients from two validation cohort showed relapse. In TCGA cohort, the 1‐ and 5‐year RFS rates were 67% and 8.8%, respectively. And the 1‐ and 5‐year OS rates were 85.5% and 43.5% in GEO50081 and 87.3% and 52.8% in GSE30219, respectively. There were 163 eligible stage I NSCLC patients enrolled with a median age of 60. One hundred thirty‐six lung adenocarcinoma account for majority of the cases. The median recurrence‐free survival time was 842 days (from 227 to 1929). Recurrence rate was 18.4% of the patients (30 of 163) in Jiangsu cohort. The 1‐ and 5‐year RFS were 90.8% and 6.3%, respectively.

### 3.2 Construction and Validation of the Recurrence Signature

**FIGURE 2:**
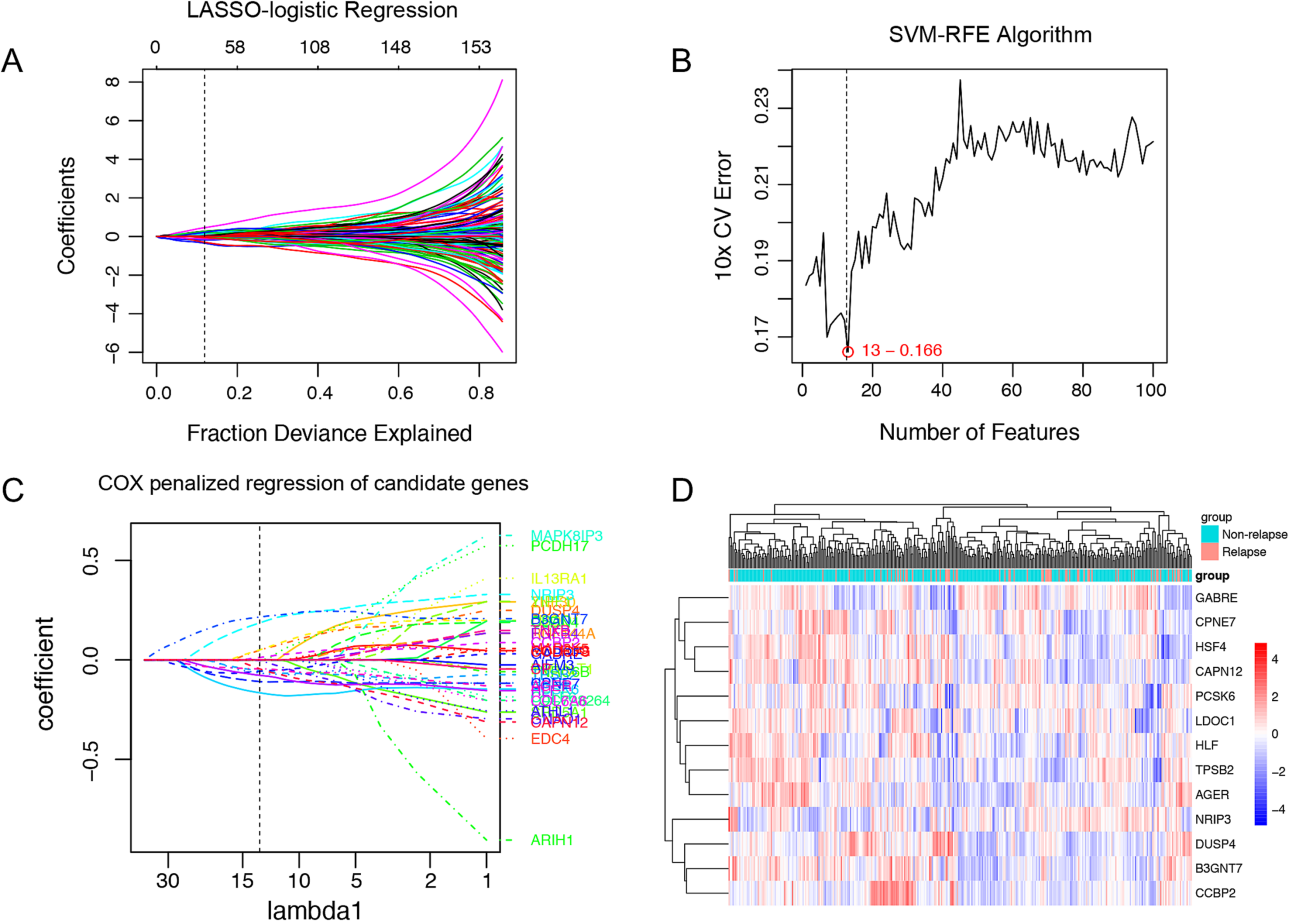
Two algorithms were used for feature selection. (A) LASSO and (B) SVM‐RFE algorithms in the discovery cohort. (C) Penalized Cox regression was used to identify the significant genes related to recurrence in training cohort. (D) Cluster analysis of incorporation of genes that were selected from previous algorithms in the discovery cohort. LASSO, least absolute shrinkage and selector operation; SVM‐RFE, support vector machine‐recursive feature elimination.

We conducted a univariable Cox regression analysis to identify 1058 genes associated with recurrence‐free survival from differential expression genes in each dataset. After analyzing the published recurrence‐associated signatures, we obtained 66 significant genes and incorporated them with significant genes from microarray datasets. LASSO analysis identified 36 candidate genes related to recurrence by the optimal lambda which was determined through 10 times cross‐validations (Figure 2A and Figure S1A). Simultaneously, another algorithm, namely, SVM‐RFE, selected 13 candidate genes through ranking the features based on their weights and eliminating the feature with the lowest weight (Figure 2B and Figure S1B). Combining the results from two algorithms, 42 candidate genes were selected, including three overlapped genes (Table S2). Then, we submitted them into penalized Cox analysis, and a risk signature was built by a 13‐mRNA with the corresponding coefficients in the training cohort (Figure 2C). A cluster plot showed the expression profile of the 13‐mRNAs (Figure 2D). Among the 13‐mRNAs, *DUSP4*, *LDOC1*, *NRIP3*, *B3GNT7*, and *CCBP2* showed positive effect in predicting the recurrence, while the rest eight genes showed negative effect in prediction, including *PCSK6*, *TPSB2*, *HSF4*, *CPNE7*, *CAPN12*, *GABRE*, *HLF*, and *AGER* (Table S3).

**FIGURE 3:**
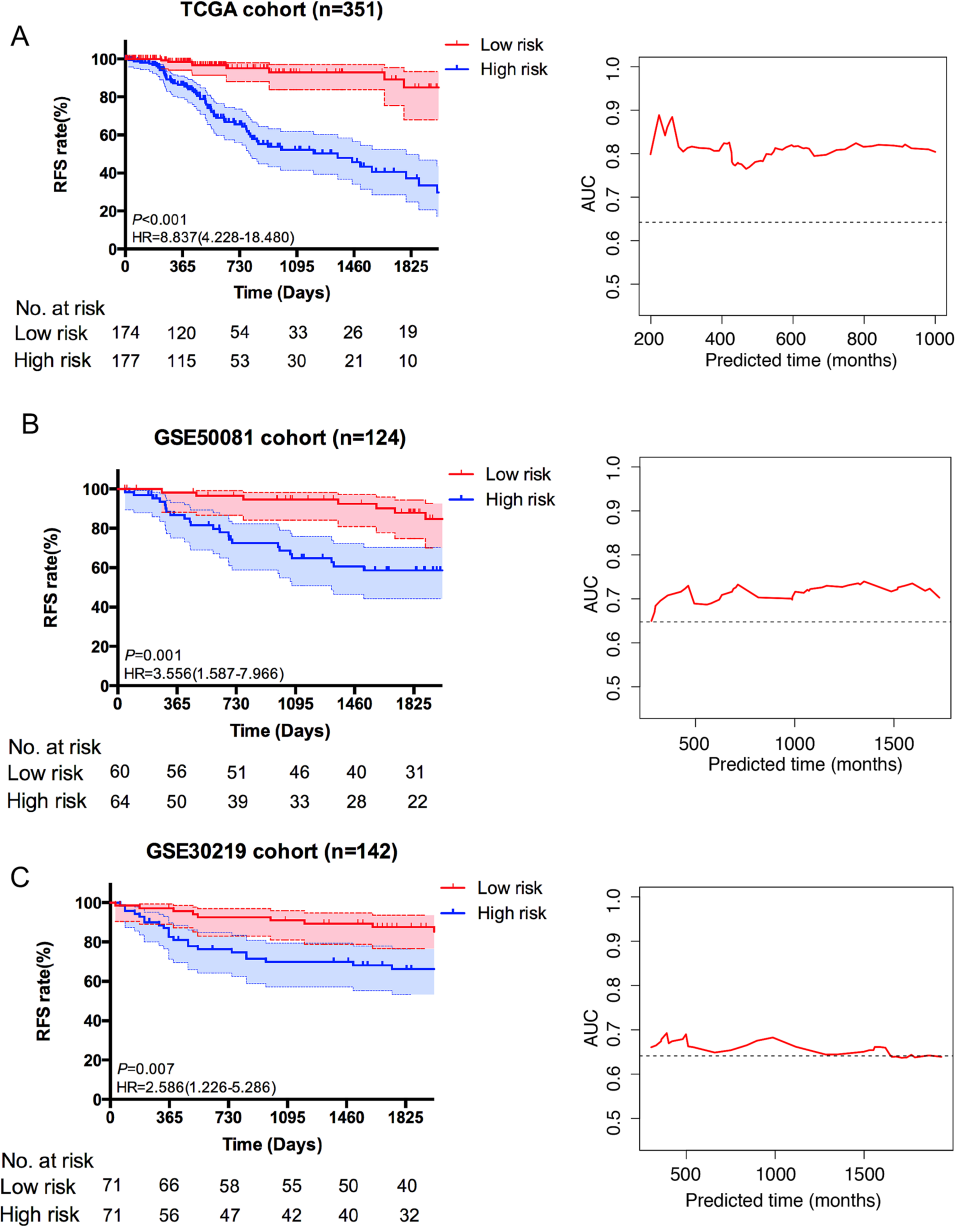
The performance of signature in predicting recurrence in the three cohorts: (A) training cohort, (B) external validation cohort 1, and (C) external validation cohort 2. AUC, area under the curve; HR, hazard ratio.

After calculating the risk score of each patient by recurrence‐related signature, we dichotomized patients in two group at the median. Kaplan–Meier (KM) survival analysis showed distinct different survival between high risk group and low risk group (hazard ratio [HR]: 8.837, 95% confidence interval: 4.228–18.480, *p* < 0.001) (Figure 3A). Prediction ability of the signature was evaluated by time‐independent ROC curve, showing well performance (AUC: 0.79 at 1‐year recurrence‐free survival).

The robustness of the 13‐mRNA signature was further confirmed in two independent cohorts (GSE50081 and GSE30219). Survival curves were remarkably different between the high risk group and the low risk group based on the signature, showing recurrence‐free survival in 51.6% and 34.3% and 56.3% and 45%, respectively (HR: 3.556, 95% CI: 1.587–7.966; *p* = 0.001 and HR: 2.586, 95% CI: 1.226–5.286; *p* = 0.007) (Figure 3B,C). Time‐independent ROC curve was used to demonstrate the predictive capability of survival prediction in two datasets. The AUCs at the 5‐year recurrence‐free survival were 0.73 in GSE50081 cohort and 0.72 in GSE30219 cohort, respectively. In addition, recurrence‐associated signature was further validated in another three cohorts that contained the 13‐mRNA expression information (GSE37745, GSE8894, and GSE31210) by a fixed effects meta‐analysis model due to their limited sample size (Figure S2). There was low heterogeneity or inconsistency across these cohorts (HR = 1.87, 95% CI: 1.29–2.70, *p* < 0.001; heterogeneity: *I* ^2^ = 0%, τ^2^ = 0, *p* = 0.37), suggesting that these data are not the result of selection bias. A strong concordance was exhibited with previous results by meta‐analysis, indicating that the recurrence pattern was a reliable and generalizable signature in predicting the recurrence of stage I NSCLC.

### 3.3 Building a Nomogram

**FIGURE 4:**
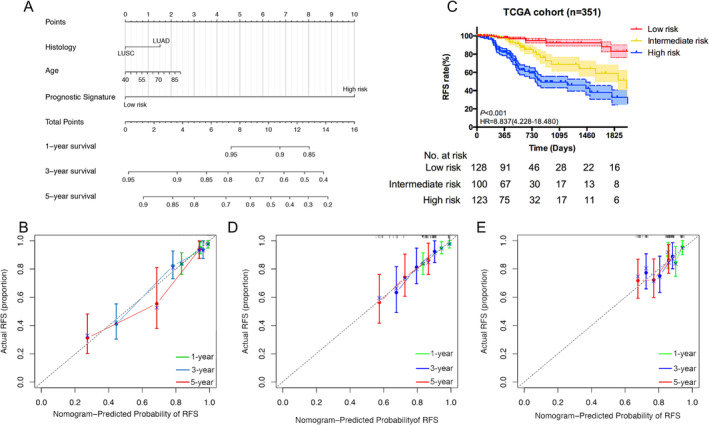
(A) Nomogram to predict the 1‐, 3‐, and 5‐year RFS. Calibration curve for RFS nomogram model in (B) TCGA cohort, (D) external validation 1 cohort, and (E) external validation 2 cohort. The dashed represents the ideal nomogram, and the green represents the 1‐year observed, blue represents the 3‐year observed, and red represents the 3‐year observed nomogram. RFS, recurrence‐free survival. (C) Kaplan–Meier curves plotting RFS of different risk subgroups stratified with nomogram score in TCGA cohort.

Univariable analysis revealed that histology and risk score were two significant risk factors for recurrence‐free survival in training cohort. After multivariable analysis, it remained as an independent risk factor (Table 2). For the purpose of clinically application, we developed a nomogram in the training cohort to individually predict the probability of recurrence by incorporating the recurrence‐related signature with age and histology selected by backward wise step method with least AIC value (Figure 4A). The concordance index (C‐index) of the nomogram was 0.737 with 95% CI ranged from 0.731 to 0.743 (*p* < 0.001), indicating the good discrimination ability. Calibration plots of 1‐, 3‐, and 5‐year illustrated the excellent accuracy of the prediction in the training cohort (Figure 4B). X‐tile software was used to generate the optimal cut‐off value by the highest *χ* ^2^ value, which categorized patients into tertiles (low risk group: 0.528–2.6, medium risk group: 2.6–11.7, and high risk group: 11.7–13.6). KM survival analysis revealed significant distinctions between each risk subgroup (HR = 8.837, 95% CI: 4.228–18.480, *p* < 0.001) (Figure 4C).

**TABLE 2:** Univariable and multivariable Cox regression analysis of training datasets.

Variables	No. of cases	Univariable		Multivariable	
		HR (95% CI)	*p*	HR (95% CI)	*p*
Training (TCGA)	351				
Age median (IQR)	68 (61–74)	1.001 (0.976–1.028)	0.925	1.011 (0.985–1.037)	0.428
Sex (man vs. women)	190/161	0.799 (0.499–1.280)	0.351	0.932 (0.578–1.502)	0.772
Smoking (smoker vs. non‐smoker)	233/118	1.003 (0.611–1.648)	0.991	1.017 (0.595–1.738)	0.952
Histology (non‐squamous vs. squamous cell carcinoma)	189/162	1.923 (1.186–3.116)	0.008*	1.369 (0.816–2.298)	0.234
Stage (IA vs. IB)	191/160	0.999 (0.622–1.606)	0.997	8.283 (3.917–17.513)	0.966
Risk score (high vs. low)	177/174	8.816 (4.216–18.436)	<0.001*	0.989 (0.603–1.623)	<0.001*
Validation 1 (GSE50081)	124				
Age median (IQR)	71 (63–76)	1.015 (0.979–1.054)	0.416	1.006 (0.970–1.043)	0.752
Sex (man vs. women)	68/56	1.550 (0.742–3.237)	0.243	1.482 (0.679–3.232)	0.323
Smoking (smoker vs. non‐smoker)	39/71^a^	0.810 (0.461–1.425)	0.465	0.634 (0.362–1.111)	0.111
Histology (non‐squamous vs. squamous cell carcinoma)	91/33	1.052 (0.484–2.286)	0.898	0.967 (0.436–2.143)	0.934
Stage (IA vs. IB)	46/78	0.336 (0.138–0.820)	0.017*	0.343 (0.138–0.848)	0.021*
Risk score (high vs. low)	64/60	3.556 (1.587–7.966)	0.002*	3.334 (1.467–7.577)	0.004*
Validation 2 (GSE30219)	142				
Age median (IQR)	62 (55–70)	1.031 (0.999–1.065)	0.060	1.024 (0.990–1.060)	0.165
Sex (man vs. women)	118/24	3.890 (0.933–6.223)	0.062	1.057 (0.404–2.764)	0.910
Smoking (smoker vs. non‐smoker)					
Histology (non‐squamous vs. squamous cell carcinoma)	86/56	1.488 (0.764–2.897)	0.242	1.789 (0.878–3.646)	0.109
Stage (IA vs. IB)					
Risk score (high vs. low)	71/71	2.521 (1.233–5.152)	0.011*	2.883 (1.353–6.140)	0.006*

Abbreviations: CI = confidence interval; HR = hazard ratio; IQR = interquartile range.

^a^ 14 cases without smoking status.

* Significant *P* value.

### 3.4 Validation of the Nomogram

**FIGURE 5:**
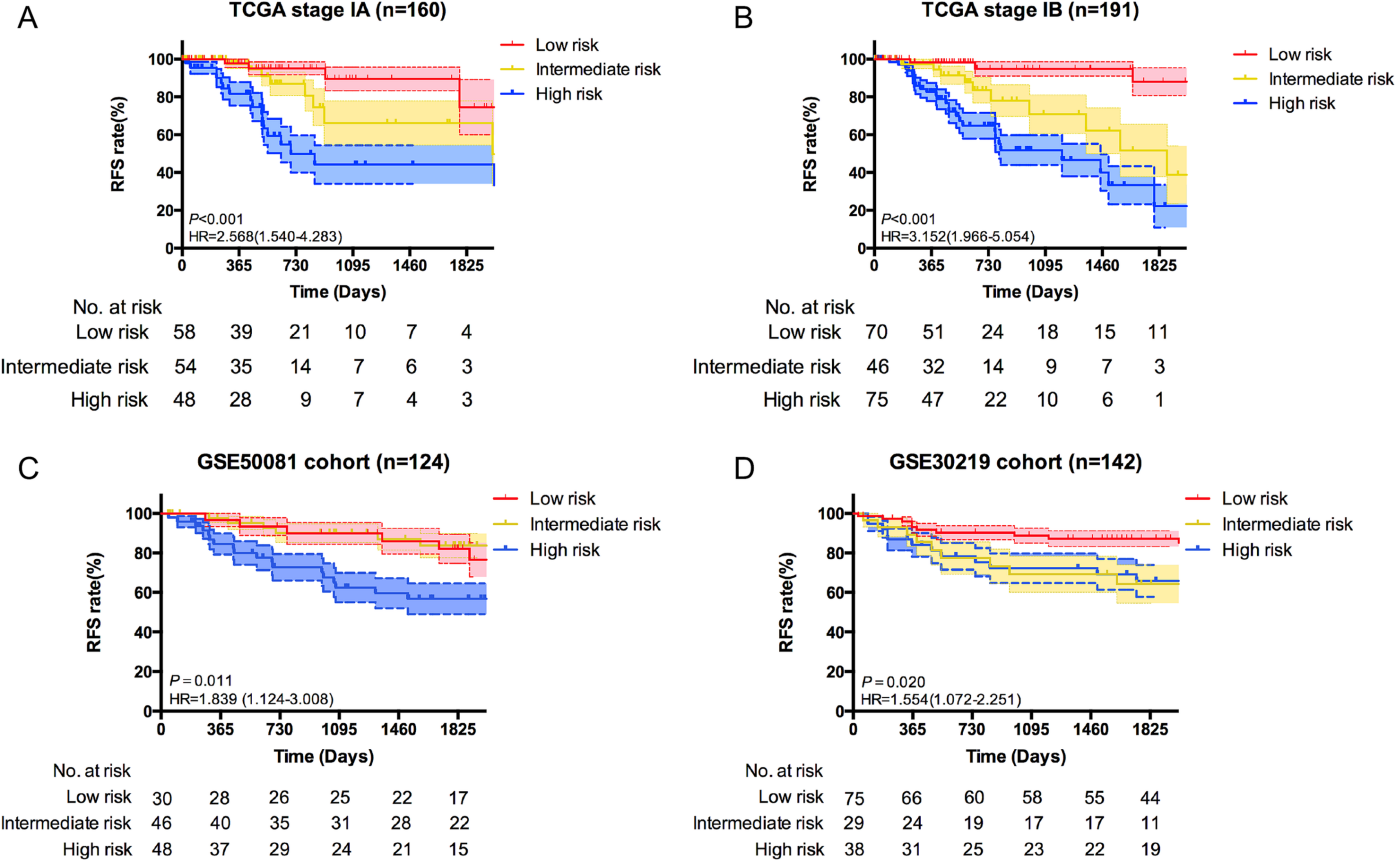
Subgroup and stratification analysis of the relapse‐related nomogram. Subgroup analysis for stage IA (A) and stage IB (B) in TCGA cohort. (C, D) Kaplan–Meier curves plotting RFS of two validation cohorts for respective nomogram score categories.

To better validate the performance of the nomogram in predicting recurrence, two previously used cohorts were utilized for further testing. The favorable calibration plots indicated that the nomogram retained the accuracy of prediction in the validation cohorts (Figure 4D,E). The discriminable ability was assessed by C‐index, which was 0.667 for the GSE50081 and 0.651 for the GSE30219 (95% CI: 0.652–0.682, *p =* 0.0002 and 95% CI: 0.637–0.665, *p =* 0.0004, respectively). Remarkably, the nomogram was separately applied to predict prognosis for stage IA and IA patients, and survival analysis yielded the significant distinctions between three subgroups, which was consistent with previous result (Figure 5A,B). Furthermore, we utilized the tertiles method in the validation cohorts. In GSE50081, low risk group and medium risk group showed significant different survival compared with high risk group (Figure 5C). But no statistical significance was achieved between low risk group and medium risk group (*p* = 0.857). While low risk group showed the better recurrence‐free survival than medium and high risk subgroup in GSE30219 (Figure 5D). However, medium risk subgroup had the similar survival with high risk subgroup (*p =* 0.939).

### 3.5 Application in a Local Cohort

**FIGURE 6:**
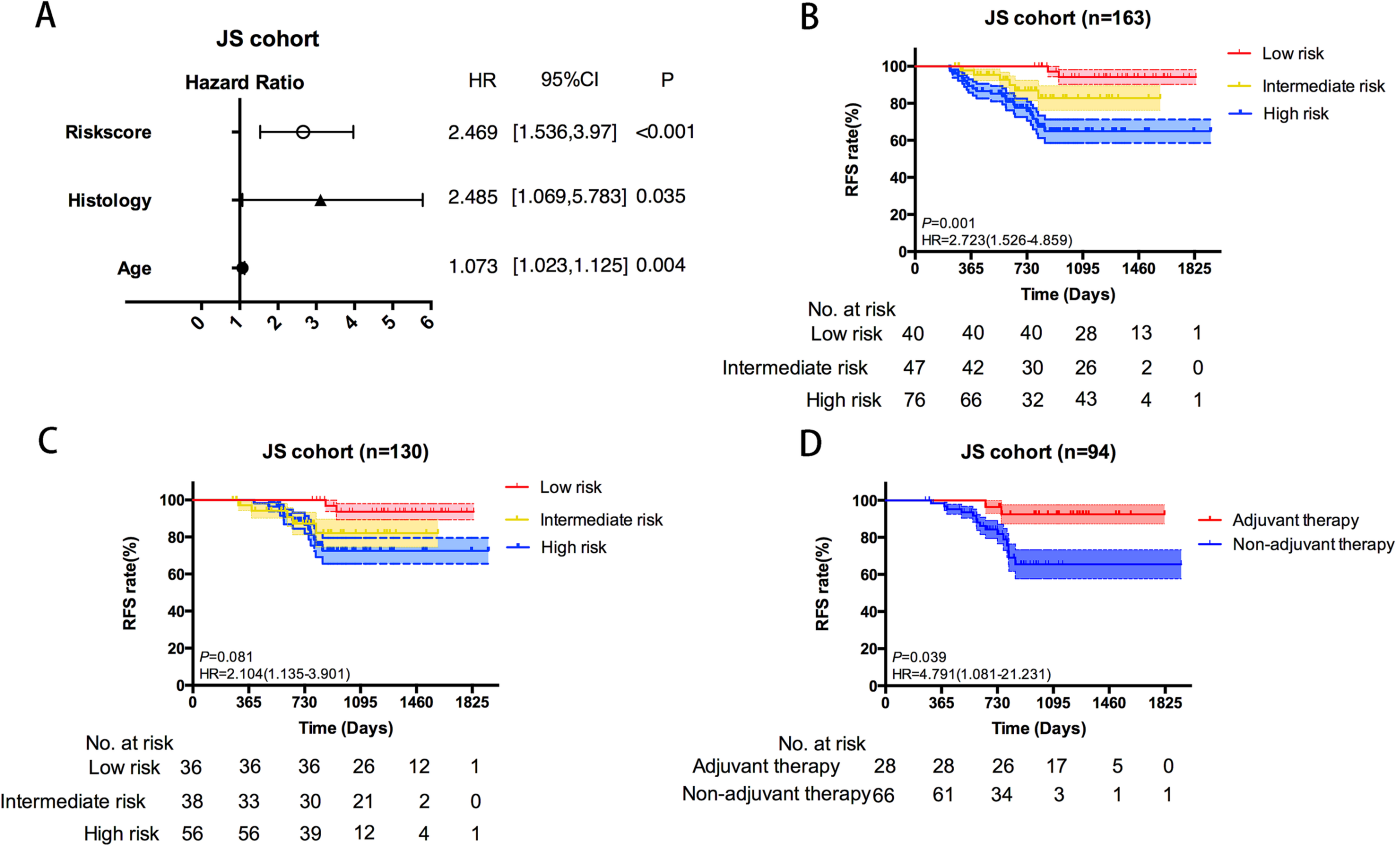
Application of the relapse‐related nomogram in local JS cohort. Hazard ratio of risk score, histology, and age in JS cohort. Subgroup analysis for stage IA (B) and stage IB (C) in JS cohort. (D) Kaplan–Meier curves plotting RFS of adjuvant therapy subgroup and non‐adjuvant therapy subgroup.

The nomogram was subsequently applied to the local cohort to test the efficiency and predicting ability. Cox analysis identified that age, histology, and risk score were independent risk factors in JS cohort (Figure 6A). In addition, high risk subgroup yielded the worst survival and three different subgroups showed obviously distinct survival curves, which was consistent with the findings from the previous results (Figure 6B). Further subgroup analysis for stage IA NSCLC patients demonstrated the same trend in survival curves, although the high risk subgroup exhibited better survival rate than it in entire group analysis (Figure 6C). Clinical information of JS cohort showed that several patients in high risk and intermedia risk subgroup receiving post‐operative adjuvant therapy due to high risk factors (micropapillary component or pleural invasion). Thus, survival analysis was conducted to compare the patients receiving adjuvant therapy with those without adjuvant therapy in order to test the discriminating ability of the risk prediction model. As expected, patients receiving adjuvant therapy showed better prognosis, indicating that adjuvant therapy could improve the survival of patients in high risk or intermedia risk subgroups (Figure 6D).

## 4 Discussion

Recurrence of stage I NSCLC is a challenging clinical issue, which shortens the survival time and reduces the effect of surgery [16]. Adjuvant therapy has been recommended for postoperative therapy for stage II or III NSCLC patients according to guidelines [3]; however, it remains controversial in stage I NSCLC. Published clinical trials have not showed a consistent survival benefit due to drug toxicity and side effect [17]. Thus, how to select the patients at high risk of recurrence is an unsolved problem in management of patients with stage I NSCLC.

Increasing investigations were devoted to seek the risk factors for recurrence of early stage NSCLC, which provided the clues for decision‐making of postoperative management [18]. Brandt et al. identified that pT stage and lymphovascular invasion were correlated with distant recurrence and declined the disease‐free survival [19]. Yu and his colleagues developed and validated a radiomics model for prediction of clinical outcome, which benefited to the choice of treatment [20]. In addition, numerous molecular signatures, based on the expression profiles, have been proposed to predict the recurrence‐free survival in recent years, which reveal the heterogeneous differences between individuals. Noro et al. proposed a two‐gene prognostic classifier to predict the recurrence for lung squamous cell carcinoma after surgical resection [21]. Shuta's study built a relapse‐related molecular signature for lung adenocarcinomas to identify patients at high risk of relapse [22]. However, as the problems of small sample size, different microarray platforms, heterogeneity from patients, and diverse range gene selection algorithms, few molecular signatures were broadly adopted in clinic for early stage lung cancer. Compared with previous studies, this study strengthened several aspects and made up the deficiencies. This novel nomogram maximized the potential of molecular biology and clinical factors. In addition, the reliability and robustness of the nomogram were tested in multiscaled cohorts from different patients and platforms, which showed well performance. As a functional tool derived from molecular biomarkers and clinical variables, it may help optimize patient care by providing better prediction of recurrence, selecting patients for postoperative adjuvant therapy, and stratifying patients in prospective clinical trials. To our knowledge, this is the first study assessing recurrence for stage I NSCLC by combining molecular signature with clinical variables.

In our preliminary work, we found 14 expression profile datasets related to recurrence from GEO database and TCGA. By checking the clinical records and the sample size, we found that TCGA contained the largest sample size and completed clinical records, GSE31210 only involved lung adenocarcinoma samples, and either of GSE41271 or GSE68465 lacked of the expression values of *TPSB2* due to different microarray platforms. Thus, we assigned TCGA into the training cohort and selected GSE50081 and GSE30129 into the validation cohort due to moderate sample size and matching clinical records. The diverse racial group and wide geographic distribution of patients made themselves representativeness and generalizability, which enhanced the reliability of the model. Candidate genes were screened by two routine algorithms in order to minimize the possibility of missing or ignoring key markers. L1 penalized Cox regression analysis, a broadly adopted method, was utilized to construct the 13‐mRNA signature from candidate genes by yielding the corresponding coefficients [14, 23]. Our 13‐mRNA signature exhibited favorable discrimination in the training and the validation cohorts, with an AUC of 0.79, 0.73, and 0.72, respectively. The cutoff values of different datasets, used to define the high risk and low risk groups by recurrence‐associated signature, were determined by the corresponding median risk scores owing to different platforms. Published studies presented evidence supporting that a series of clinical variables, such as age, histology, and differentiation, were associated with recurrence‐free survival of early stage NSCLC [10]. Therefore, we considered the clinical variables and constructed a nomogram by incorporating clinical variables with our molecular signature to provide an easy‐to‐use tool for clinicians, showing good calibration and discrimination in the training and validation cohorts. Univariable regression analysis revealed that histology showed statistically significant results in the training cohorts. However, after adjusting with the 13‐mRNA signature, it was not significant, which might be related to the respective small sample size. And our meta‐analysis of the entire cohort revealed that age and histology were two key variable associated with recurrence‐free survival (Figure S3). Backward wise step method demonstrated that age, histology, and signature were eligible variables with the least AIC value, which could be incorporated into the nomogram. Validation analysis confirmed the reliability and generalization of the nomogram. Tertile‐stratified method allowed the remarkably distinctions between survival curves. Notably, we found no statistically significant differences between low and medium risk groups in GSE50081 and medium and high risk groups in GSE30219. This might be the lower samples in low risk group in GSE50081 and lower samples in medium risk group, which could not discriminate themselves from other groups. In addition, we considered that the TNM stage system was another significant parameter in this analysis; thus, we compared the efficiency of our predicting model with the TNM stage system. The result showed that our model performed well in both TCGA and GSE50081 datasets, while no information about stage was found in GSE30219 dataset (Figure S4).

Heterogeneity of immune infiltration was vital for progression of early stage NSCLC. CIBERSORTx online tool was used to estimate immune infiltration. The immune cell infiltration level different subpopulations of eight immune infiltration cells between different risk levels were presented in Figure S5, which was revealed weakly to moderately correlated, especially in the relationship between risk score and CD4+ T cells or T reg cells.

There are some limitations of this study that should be mentioned. First of all, this was a multiscaled study based on the datasets from GEO and TCGA, but prospective studies are required to further validate our finding. In addition, several significant clinical variables were not recorded in some datasets, which hampered the accuracy of our model. For example, the FDG value of PET/CT was an important factor for early stage NSCLC. Based on current resources and capabilities, we were unable to include this issue into our analysis. Furthermore, different platforms of the datasets hindered the integrated analysis of these datasets, which reduced the power of the model.

## 5 Conclusion

In this study, we developed a reliable and robust nomogram that could be reproducible in ethnically and geographically diverse cohorts. It will provide classification of patients at high risk for recurrence is important for identifying those who may benefit from adjuvant chemotherapy and optimize the design of prospective clinical trials.

## Author Contributions

Q.C., L.W., and Q.M. designed the study. R.B., F.Z., and B.P. conducted the bioinformatics analysis. J.Z., L.W., and Q.C. downloaded public datasets and provided clinical support. L.W. and R.B. wrote the article.

## Ethics Statement

The study was approved by the Regional Ethics Committee at Xuzhou Central Hospital.

## Consent

The consent was obtained from all patients.

## Conflicts of Interest

The authors declare no conflicts of interest.

## Supporting information


**Table S1.** Published signatures or genes related to recurrence of early stage NSCLC.
**Table S2.** Candidate genes were selected using the LASSO and SVM‐RFE algorithms between recurrent and non‐recurrent samples.
**Table S3.** Coefficients of candidate genes in recurrence associated signature.

**Figure S1.** Cross‐validation for tuning parameter selection in the LASSO (A) and penalized Cox regression (B). The dashed lines were drawn at the optimal values. The optimal tuning parameter lambda was 14.024 with ten‐time cross‐validation for penalized cox regression.

**Figure S2.** Forest plot of the prognostic signature in three independent cohorts with RFS outcome. CI, confidence interval.

**Figure S3.** Forest plots of the clinical features in TCGA, GSE50081, and GSE30219. (A) age, (B) histology, (C) sex, (D) stage, (E) smoking, and (F) risk score. CI, confidence interval.

**Figure S4.** ROC curves elucidated the stage and subgroup analysis of TCGA and GSE50081.

**Figure S5.** Subgroup of immune cell infiltration level between different risk levels.

**Data S1.** Supplementary Information.

## Data Availability

The data that support the findings of this study are available in TCGA at https://www.cancer.gov/ccg/research/genome‐sequencing/tcga. These data were derived from the following resources available in the public domain: GEO number: GSE30219, https://www.ncbi.nlm.nih.gov/geo/query/acc.cgi?acc=GSE30219.
