# Supplementary material for: A Nomogram for Predicting Recurrence in Stage I Non‐Small Cell Lung Cancer
Source: Clin Respir J. 2024 Nov 24;18(11):e70022. doi: 10.1111/crj.70022 (PMC11586294; doi:10.1111/crj.70022)
Supplement: Supplementary file 1 — Table S1. Published signatures or genes related to recurrence of early stage NSCLC. Table S2. Candidate genes were selected using the LASSO and SVM‐RFE algorithms between recurrent and non‐recurrent samples. Table S3. Coefficients of candidate genes in recurrence associated signature. [file CRJ-18-e70022-s006.docx]

Table S1. Published signatures or genes related to recurrence of early stage NSCLC.

| Author & ref. | Year | Sample size | race | genes |
| --- | --- | --- | --- | --- |
| Noro et al | 2017 | 212 | Japan/Norway | \| *DUSP6,* \| *ACTN4* \| \| --- \| --- \| |
| Robles et al | 2015 | 73 | Japan/Norway | *BRCA1, XPO1, HIF1A, DLC1* |
| Lu et al | 2012 | 142 | Korea/Australia | *AU148154, B4GALT1, CGB, CHST12, CLEC11A, COL2A1, CYP2A6, DENND1A, DIO1, DOCK6, EPHB6, FZD9, GLE1, GTF3C2, INF2, KDM4B, SIK3, GREB1L, KLK5, KRT81, LENEP, MYOG, NFKBIL1, NLRP2, FEZ2, OCA2, PADI3, RPRM, SH3YL1, SLC27A2, SLC35F5, SNAPC2, SPTBN2, STRN3, SUSD4, TCF3, TET3, THBS1, TRIM34, TRIM46, TRIP11, CELSR1, UBE2D4, UBXN4, VKORC1, ZBTB7B, ZNF365, MUC5AC, FGFR2* |
| Mitra et al | 2011 | 27 | USA | *DBN1, CACNB3, FLAD1, CCND2* |
| Lee et al | 2008 | 138 | Korea | *CALB1, MMP7, SLC1A7, GSTA1, CCL19, IFI44* |
| Larsen et al | 2007 | 51 | Australia | *ABCA1, ABCC5, ACOT2, AMDHD1, ARFGEF2, ASB7, ATP6N1A, BITE, BRWD1, C11orf10, C14orf155, C16orf57, C18orf15, C1orf112, C1orf86, C1QC, C3orf9, CABP3, CAPN3, CDH20, CES1, CHD2, CLPX, COX15, CRISP3, CSMD2, CTSS, DCLRE1C, DOCK2, DYM, EML2, ENPP6, EVPL, FAM11A, FLJ21836, FLJ31943, FRMD4A, FTL, FXYD3, GOLPH3L, HCDI, HERC2P7, HRASLS, HSPG2, IL7R, IMP4, ITCH, KCNMA1, KIAA0372, KLHL3, LMLN, LOC129285, LOC144438, LOC642776, LOC92482, LRIG2, MAF, MGC16037, NP, NR1D2, P38IP, PDCD10, PDZD2, PGBD5, PIN1L, POLR3G, PP35, PPM1B, PRLR, PRO1051, PTPLA, PTPN21, PTPRC, RAB11FIP4, RDH14, RHBDF1, RP11-308B5.5, RPL15, RSPRY1, SCGB1D1, SCGB2A1, SEL1L, SFRS10, SLC37A1, STEAP1, STX11, TIAM1, TMEM47, TPBG, TRIM22, TRPC5, TRPM1, TRSPAP1, ZNF277, ZNF292, ZNF302* |
| Tomida | 2009 | 117 | Japan | *TM2D2, RPP40, PGRMC1, C11orf73, RNF170, FARSLB, IFT52, PRPF4B, CXorf56, API5, PICALM, VBP1, PSMD10, COIL, CDC91L1, SMARCE1, LIME1, STK38, MGC12966, SRP9, NOL11, OR11A1, UMPS, TADA2L, TADA2L, UBE2Q2, PSMD12, ZBTB9, EIF2S1, CBWD3, C6orf62, ARMC8, ZNF525, RANBP9, VDAC3, MTERFD1, ELF5, FIP1L1, CXorf56, TMEM14B, CKAP5, COL9A1, ZBTB33, LOC494150, HNRPH3, UBE2W, HSD17B12, PRPF4B, UBE2V2, RBM34, SRP9, RBMX, CACYBP, BRWD1, HNMT, RIOK1, GOLGA7, E2F6, PSMD12, PDCL3, EIF2S2, TIPRL, SEC22A, METTL2A* |

Table S2. Candidate genes were selected using the LASSO and SVM-RFE algorithms between recurrent and non-recurrent samples.

| Gene ID | β value | Method |
| --- | --- | --- |
| POLR3G | -0.2067 | LASSO |
| MAP2K5 | -0.1938 | LASSO |
| EDC4 | -0.1154 | LASSO |
| DUSP4 | -0.1094 | LASSO |
| MPV17 | -0.1136 | LASSO |
| RNF144A | -0.0732 | LASSO |
| ZNF30 | -0.0598 | LASSO |
| LDOC1 | -0.0557 | LASSO |
| ARPC4 | -0.0531 | LASSO |
| IL13RA1 | -0.0483 | LASSO |
| B4GALT1 | -0.0381 | LASSO |
| YIPF1 | -0.0328 | LASSO |
| SRD5A1 | -0.0301 | LASSO |
| TNRC6B | -0.0295 | LASSO |
| PCDH17 | -0.0239 | LASSO |
| ARIH1 | -0.0138 | LASSO |
| CLDN4 | -0.0107 | LASSO |
| CSAD | -0.0062 | LASSO |
| LOC728264 | 0.0129 | LASSO |
| CELF6 | 0.0134 | LASSO |
| MAPK8IP3 | 0.0154 | LASSO/SVM-RFE |
| NRIP3 | 0.0197 | LASSO |
| PCSK6 | 0.0333 | LASSO |
| TPSB2 | 0.0525 | LASSO |
| HSF4 | 0.0676 | LASSO |
| B3GNT7 | 0.0787 | LASSO/SVM-RFE |
| CPNE7 | 0.1203 | LASSO |
| AIFM3 | 0.1269 | LASSO |
| GABRE | 0.1962 | LASSO |
| ATHL1 | 0.2594 | LASSO/SVM-RFE |
| GNAO1 | 0.2739 | LASSO |
| SPTBN5 | 0.2837 | LASSO |
| FGFR4 | -0.1334 | SVM-RFE |
| HLF | 0.2101 | SVM-RFE |
| PTGDS | -0.1019 | SVM-RFE |
| CCBP2 | 0.3021 | SVM-RFE |
| COL6A6 | -0.1043 | SVM-RFE |
| AGER | 0.0293 | SVM-RFE |
| TNXB | 0.0192 | SVM-RFE |
| CP | -0.1346 | SVM-RFE |
| CAPN12 | 0.1948 | SVM-RFE |
| CCDC78 | -0.0321 | SVM-RFE |

Table S3. Coefficients of candidate genes in recurrence associated signature.

| Candidate genes | Coefficient |
| --- | --- |
| DUSP4 | 0.0434 |
| LDOC1 | 0.0456 |
| NRIP3 | 0.1808 |
| PCSK6 | -0.1626 |
| TPSB2 | -0.0530 |
| HSF4 | -0.0635 |
| B3GNT7 | 0.2137 |
| CPNE7 | -0.1075 |
| CAPN12 | -0.0389 |
| GABRE | -0.0587 |
| HLF | -0.0757 |
| CCBP2 | 0.0102 |
| AGER | -0.0449 |
