# Supplementary figures and images for: A Nomogram for Predicting Recurrence in Stage I Non‐Small Cell Lung Cancer
Source: Clin Respir J. 2024 Nov 24;18(11):e70022. doi: 10.1111/crj.70022 (PMC11586294; doi:10.1111/crj.70022)

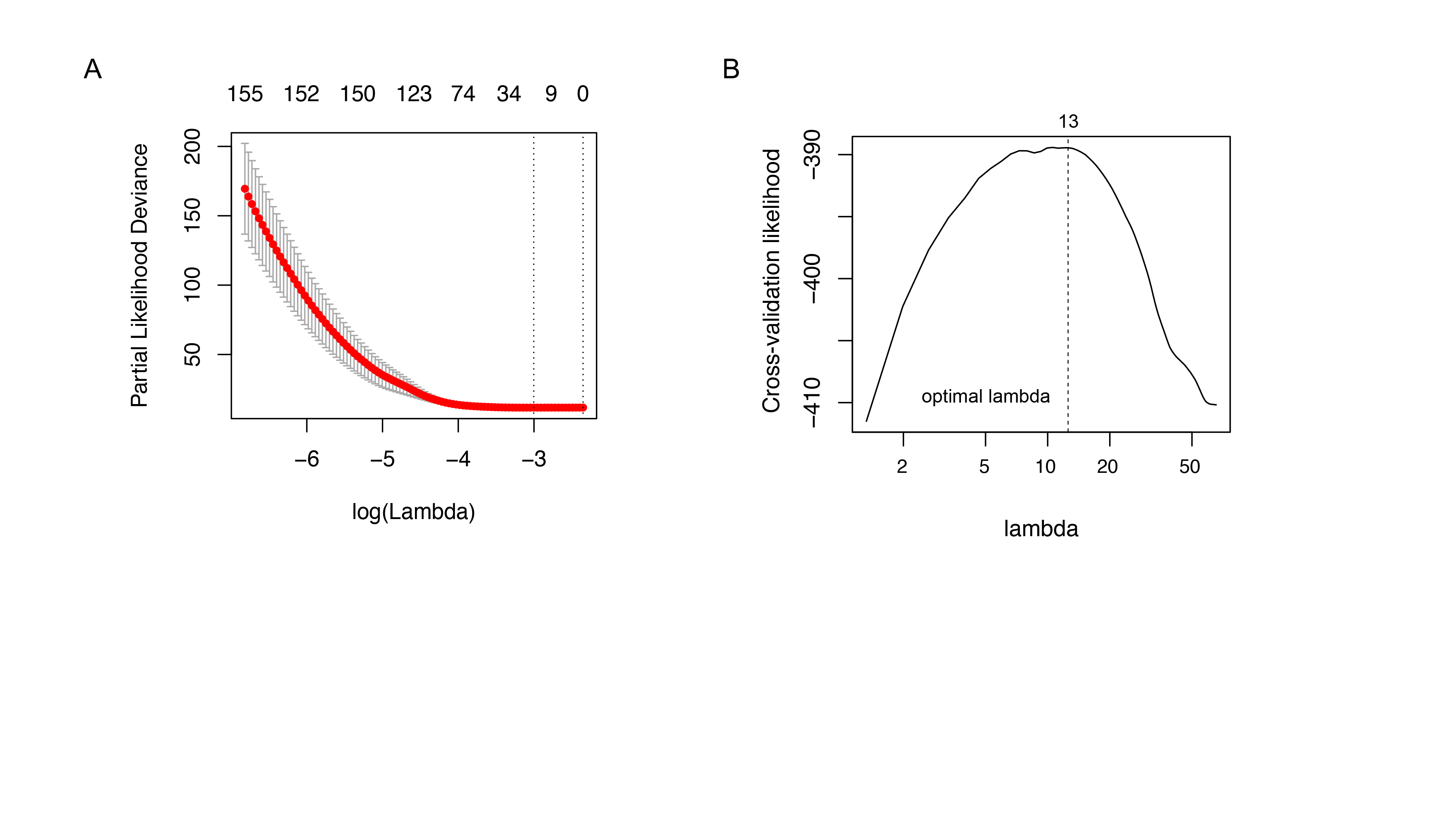

Supplement: Supplementary file 2 — Figure S1. Cross‐validation for tuning parameter selection in the LASSO (A) and penalized Cox regression (B). The dashed lines were drawn at the optimal values. The optimal tuning parameter lambda was 14.024 with ten‐time cross‐validation for penalized cox regression. [file CRJ-18-e70022-s002.tif]

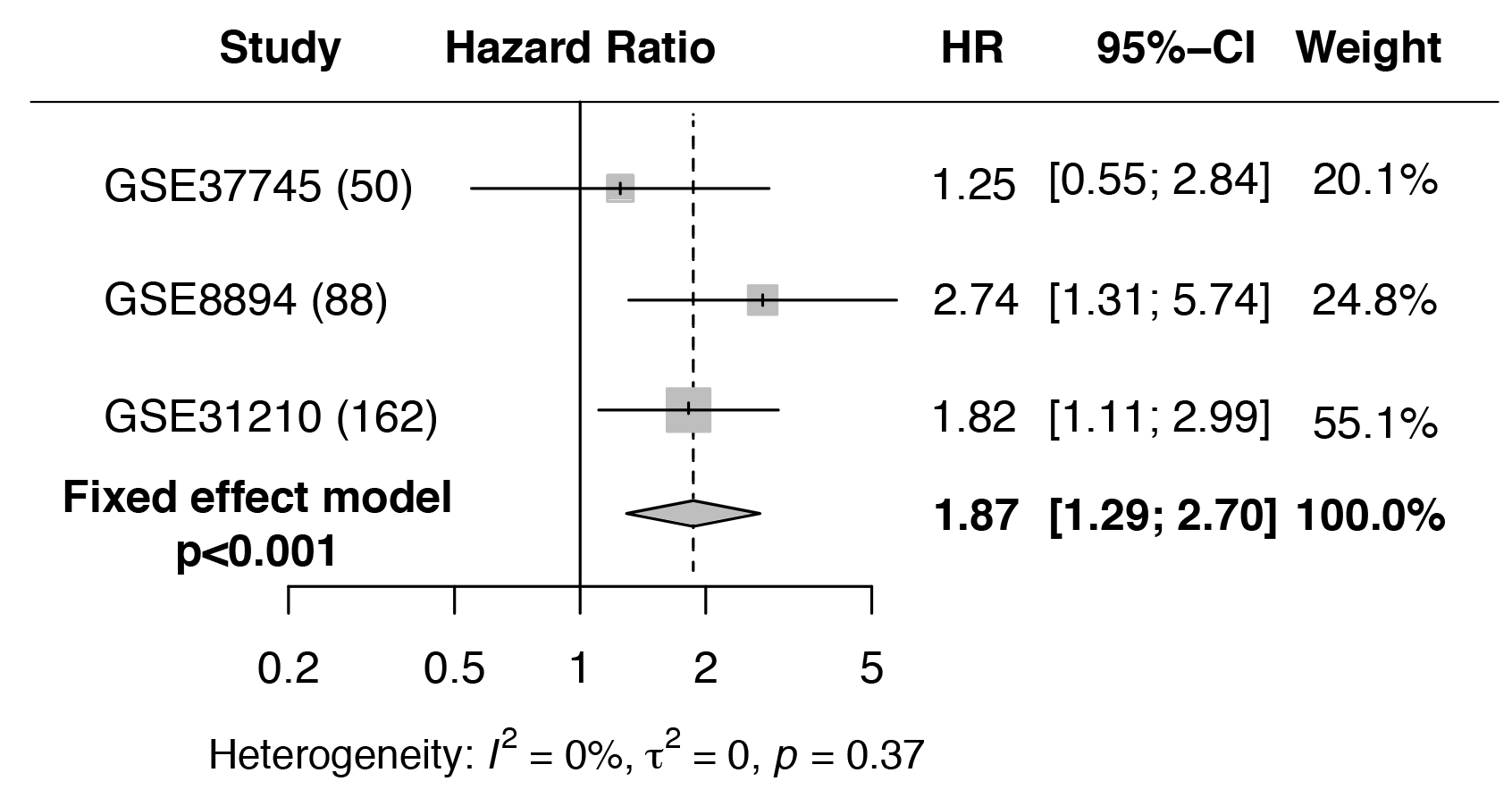

Supplement: Supplementary file 3 — Figure S2. Forest plot of the prognostic signature in three independent cohorts with RFS outcome. CI, confidence interval. [file CRJ-18-e70022-s004.tif]

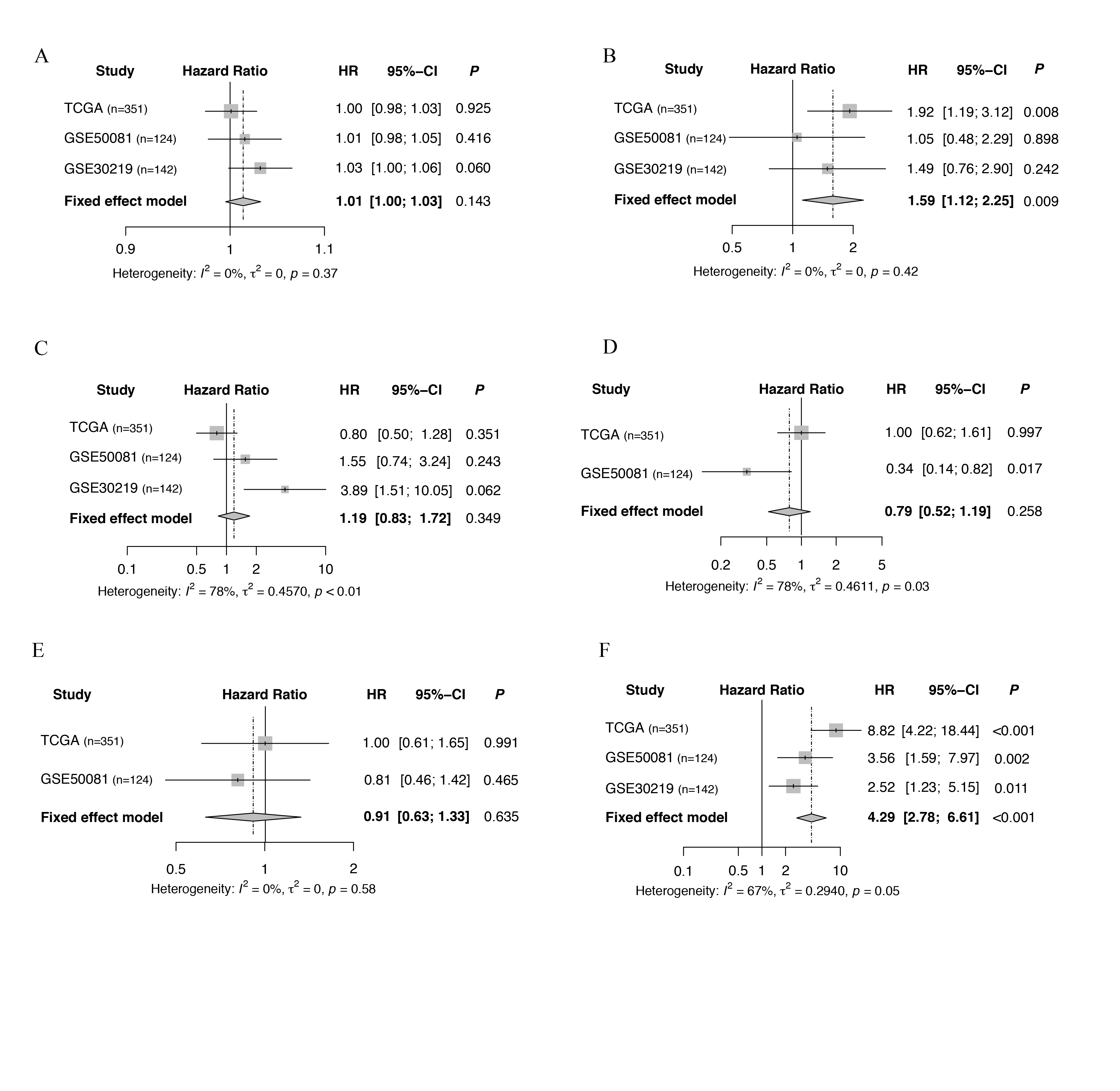

Supplement: Supplementary file 4 — Figure S3. Forest plots of the clinical features in TCGA, GSE50081, and GSE30219. (A) age, (B) histology, (C) sex, (D) stage, (E) smoking, and (F) risk score. CI, confidence interval. [file CRJ-18-e70022-s001.tif]

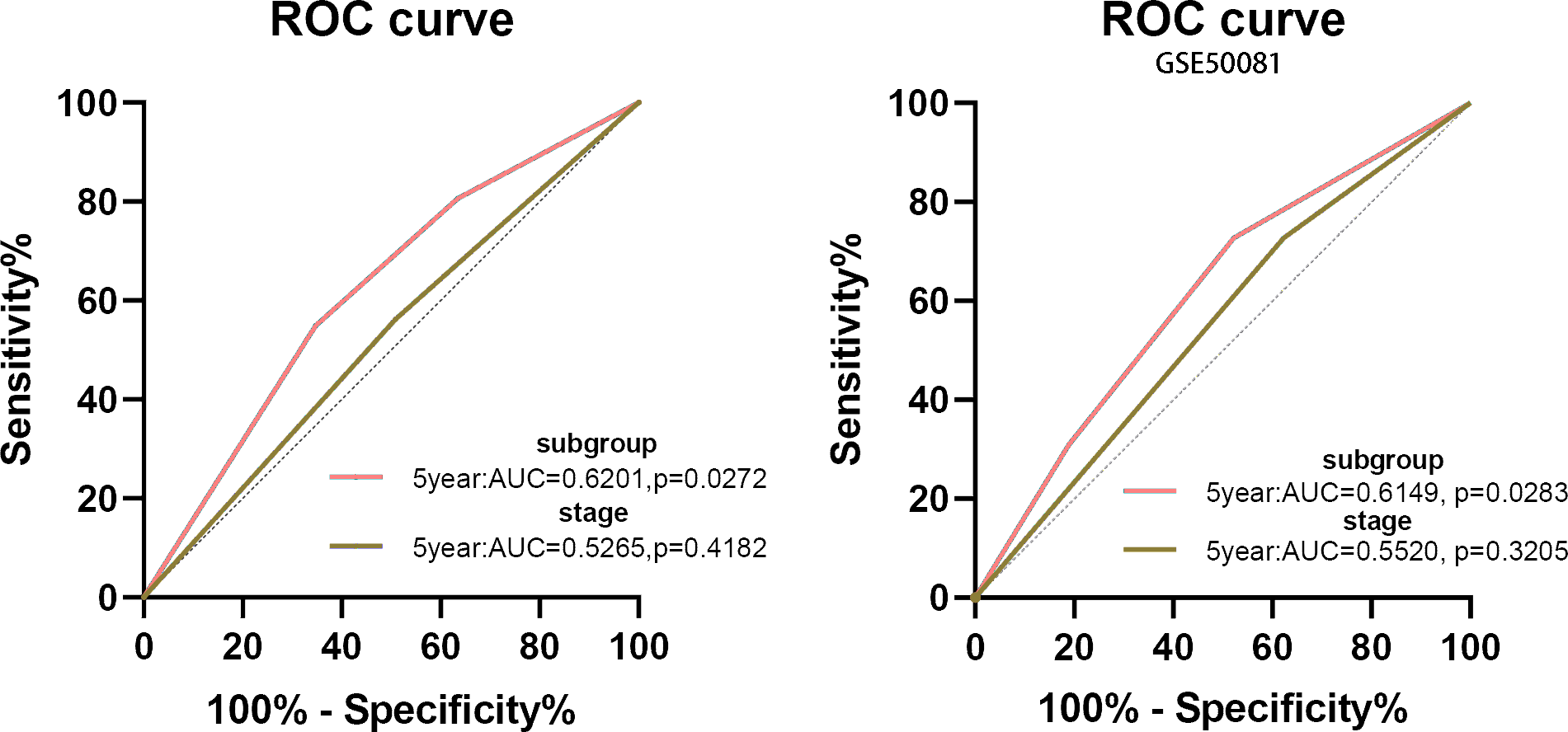
TCGA

Supplement: Supplementary file 5 — Figure S4. ROC curves elucidated the stage and subgroup analysis of TCGA and GSE50081. [file CRJ-18-e70022-s005.docx]

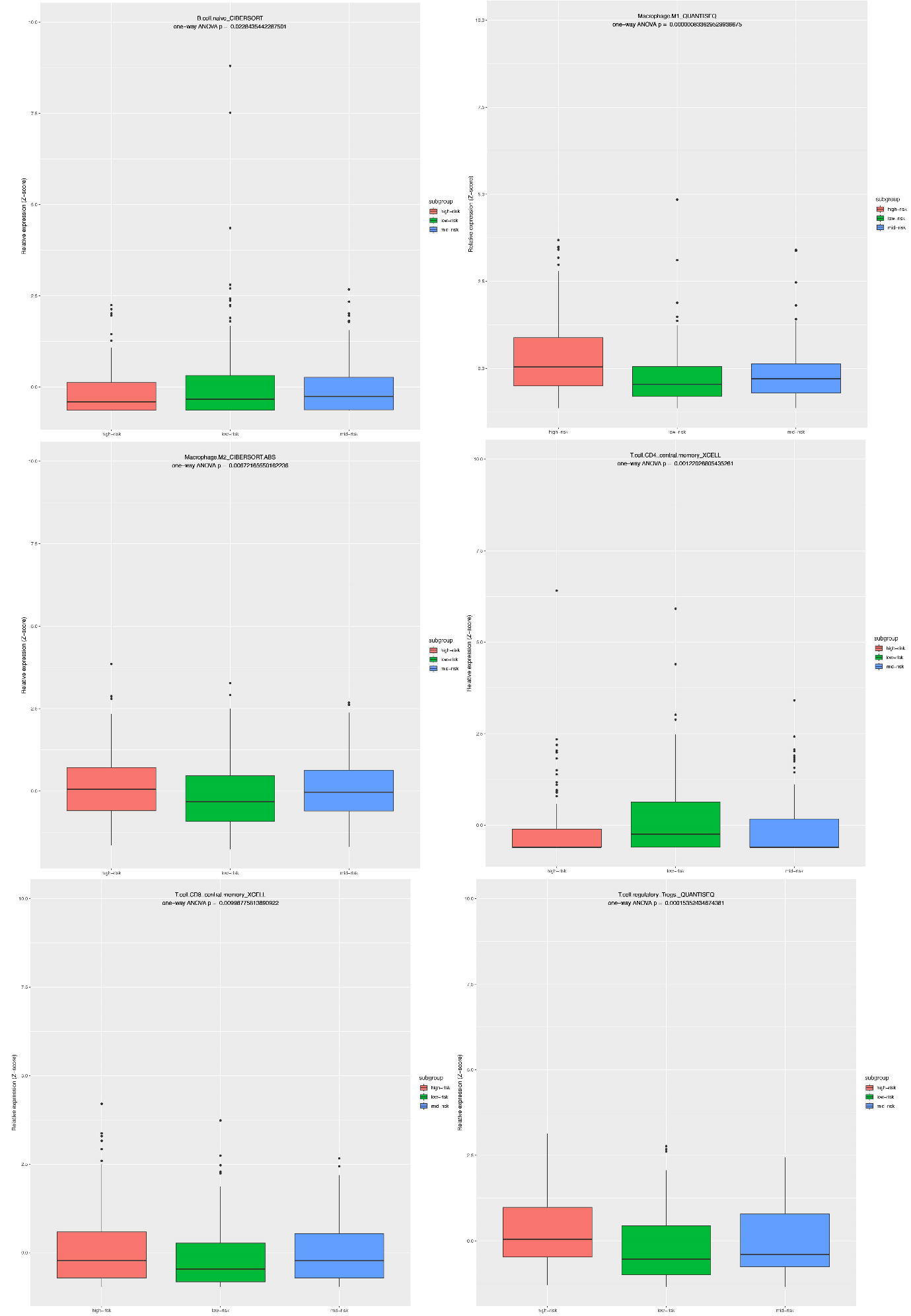

Supplement: Supplementary file 6 — Figure S5. Subgroup of immune cell infiltration level between different risk levels. [file CRJ-18-e70022-s007.docx]
