# Supplementary material for: A Nomogram for Predicting Recurrence in Stage I Non‐Small Cell Lung Cancer
Source: Clin Respir J. 2024 Nov 24;18(11):e70022. doi: 10.1111/crj.70022 (PMC11586294; doi:10.1111/crj.70022)
Supplement: Supplementary file 7 — Data S1. Supplementary Information. [file CRJ-18-e70022-s003.docx]

Figure Legend:

Figure S4. ROC curves elucidated the stage and subgroup analysis of TCGA and GSE50081.

Figure S5. Subgroup of immune cell infiltration level between different risk levels.
